# The UV filtering potential of drop-casted layers of frustules of three diatom species

**DOI:** 10.1038/s41598-018-19596-4

**Published:** 2018-01-17

**Authors:** Yanyan Su, Torben A. Lenau, Emil Gundersen, Jacob J. K. Kirkensgaard, Christian Maibohm, Jérôme Pinti, Marianne Ellegaard

**Affiliations:** 1 Department of Plant and Environmental Sciences, University of Copenhagen, Thorvaldsensvej 40, 1871 Frederiksberg, Denmark; 2 Department of Mechanical Engineering, Technical University of Denmark, Produktionstorvet, Building 426, 2800 Kongens Lyngby, Denmark; 3 Niels Bohr Institute, University of Copenhagen, 2100 Copenhagen, Denmark; 4 International Iberian Nanotechnology Laboratory, Avenida Mestre José Veiga s/n, 4715-330 Braga, Portugal; 5 DTU Aqua, National Institute of Aquatic Resources, Technical University of Denmark, Kemitorvet, building 202, 2800 Kongens Lyngby, Denmark

## Abstract

Diatoms are in focus as biological materials for a range of photonic applications. Many of these applications would require embedding a multitude of diatoms in a matrix (e.g. paint, crème or lacquer); however, most studies on the photonic and spectral properties of diatoms frustules (silica walls) have been carried out on single cells. In this study, for the first time, we test the spectral properties of layers of frustules of three diatom species (*Coscinodiscus granii*, *Thalassiosira punctifera* and *Thalassiosira pseudonana*), with special focus on transmission and reflectance in the UV range. The transmittance efficiency in the UV A and B range was: *T. pseudonana* (56–59%) >*C. granii* (53–54%) >*T. punctifera* (18–21%) for the rinsed frustules. To investigate the underlying cause of these differences, we performed X-ray scattering analysis, measurement of layer thickness and microscopic determination of frustule nanostructures. We further tested dried intact cells in the same experimental setup. Based on these data we discuss the relative importance of crystal structure properties, nanostructure and quantity of material on the spectral properties of diatom layers. Characterization of the UV protection performance of layers of diatom frustules is of central relevance for their potential use as innovative bio-based UV filters.

## Introduction

The pursuit of new materials for protecting against UV irradiance is ongoing due to the high demand of UV protection accompanying the continuous increase of UV exposure on the earth^1^ and the limitations of conventional UV blockers^2,3^, which are synthesized compounds, including organic UV absorbers containing phenolic groups with intramolecular hydrogen bonds^4^ and inorganic metal oxide nanoparticles such as TiO_2_, ZnO and CeO_2_ with UV light scattering and absorption properties^5^. A cost-effective, facile and environmental friendly biomaterial that could be used as UV filter is thus given high priority.

Diatoms, as one of the most successful groups of organisms on earth with huge biogeochemical and ecological significance, are present in almost all aquatic and moist environments. Apart from their enormous ecological importance, diatoms are unique in their ornate petri dish like silica cell covering (the frustule) exhibiting intricate and species-specific patterns, joined by one or several girdle bands^6^. Chambers and pores with precise and repeated nanometer-scale features, are regularly distributed in both the cell wall of valves and girdle bands^7^. The ability of diatoms to manipulate silicon to fabricate intricate and diverse structures at the nano- to millimeter scale exceeds that of present-day human material engineering^8^. In a few cases diatoms have been shown to also incorporate calcium-based structures^9^. It has been suggested that diatoms optimize the 3D architecture of their frustule to maximize visible light utilization and minimize the damage from UV irradiation during their evolution^10^. It has further been hypothesized that the frustule plays a role in protecting diatom cells against UV irradiation, thereby functioning as a biological UV shield^11–13^.

Numerical simulations have provided evidence that both centric (with a radial symmetry of the valves) and pennate (with bilaterally symmetrical valves) frustules could act as a filter in the UV range, with centric diatoms more effective than pennate^13^. The possibility to form photonic band gaps or Bragg scattering, preventing specific wavelengths from propagating through the valve, has been calculated for single frustules of the centric diatom *Melosira variance*^14^. The propagation of visible light in both valves and girdle bands of the centric diatom *Coscinodiscus granii* has been simulated^7,15^. De Tommasi *et al*.^16^ investigated transmission of light (from green to red, 532, 557, 582 and 633 nm) through single valves of the centric diatom *Coscinodiscus wailesii* both numerically and experimentally. The optical properties of single valves of the centric diatom *Arachnoidiscus* sp. have been documented by both simulation and experiments in the ultraviolet and visible range^10^. However, all of these studies are based on single valves and most of them are confined to simulation studies. The overall performance of a layer of diatom-based material in light propagation, which is very important for diatom-based photonic applications, has never been reported.

In this study, three centric diatom species, *Coscinodiscus granii*, *Thalassiosira pseudonana* and *Thalassiosira punctifera*, were selected due to their range of different morphological characteristics as reported in literature^17^. These characteristics, in particular the size and periodicity of the larger pores (foramen) in the frustule, have been hypothesized to be central for the photonic characteristics of the frustule^11^ and we wanted to see if these differences in morphology were also central to optical properties of a random layer. Another reason for the selection of the genus *Coscinodiscus* is that it has been explored intensively for its potential in optical applications^13,15,18^ due to its large and flat valve, and we therefore have an extensive body of research on single valves to compare with. *Thallassiosira pseudonana* was in part selected because it is one of the few diatom species which have been whole-genome-sequenced^19^, and it therefore is potentially a model for genetic modification of frustule properties. For the first time, the transmittance, reflectance and absorbance of visible and UV light by layers of frustules drop-casted onto a surface were investigated and factors that might influence light propagation, especially with regard to UV protection efficiency, were analyzed. We tested the performance of both intact cells with cell content and separated valves, rinsed of organic material, and discuss the respective properties of these. These results offer new information to expand the properties of single frustule valves to possible applications requiring layers of frustules (e.g. in a matrix), with particular emphasis on potential for UV protection.

## Results

### Coverage rate

The mean coverage rate of the single and double drop-casted layers is above 91% for both dried intact cells and rinsed frustules for all three species (Table S1 and Fig. S1 in supplementary information).

### UV transmission and reflection efficiencies by the rinsed frustules of three diatom species

**Figure 1:**
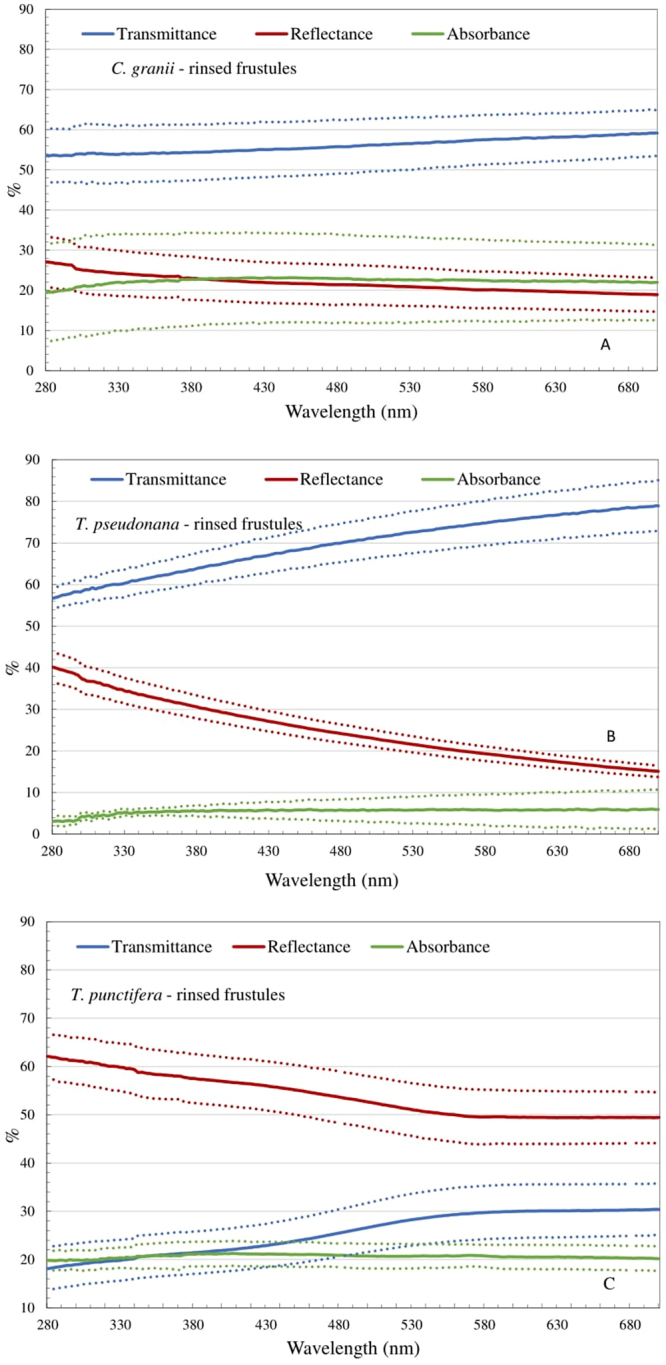
Spectral transmittance (blue), reflectance (red) and absorbance (green) for a coating of rinsed *C. granii* (**A**), *T. pseudonana* (**B**) and *T. punctifera* (**C**) frustules. The dotted lines represent the mean value± the standard deviation. The reference spectrum is the UV-transparent quartz silica microscope glass plate with milli q water on top of it. All the values are normalized to 100% coverage.

Spectra from 280 to 700 nm of layers of rinsed frustule of each of the three tested diatom species are presented in Fig. 1, which shows the different patterns in transmittance, reflectance and absorption.

For all three species, transmission efficiency increased with increasing wavelength, steeper for *T. pseudonana* and *T. punctifera* than *C. granii* (Fig. 1). The transmittance efficiency in the UV range (280–400 nm) was 18–21%, 53–54% and 56–59% for *T. punctifera*, *C. granii* and *T. pseudonana* (Fig. 1), respectively. The opposite trend was seen in reflectance which for all three species decreased with increasing wavelength. A slightly higher reflectance in UV-B (below 315 nm) range than in UV-A range (315 to 400 nm) was observed for all three species. Altogether, *T. punctifera* frustules have the lowest UV transmittance and highest UV reflectance. Absorption levels (calculated rather than measured) were relatively constant throughout the whole measured spectrum at c. 20% for *C. granii* and *T. punctifera* and 5% for *T. pseudonana* (Fig. 1).

### UV transmission and reflection efficiencies of dried intact cells of three diatom species

**Figure 2:**
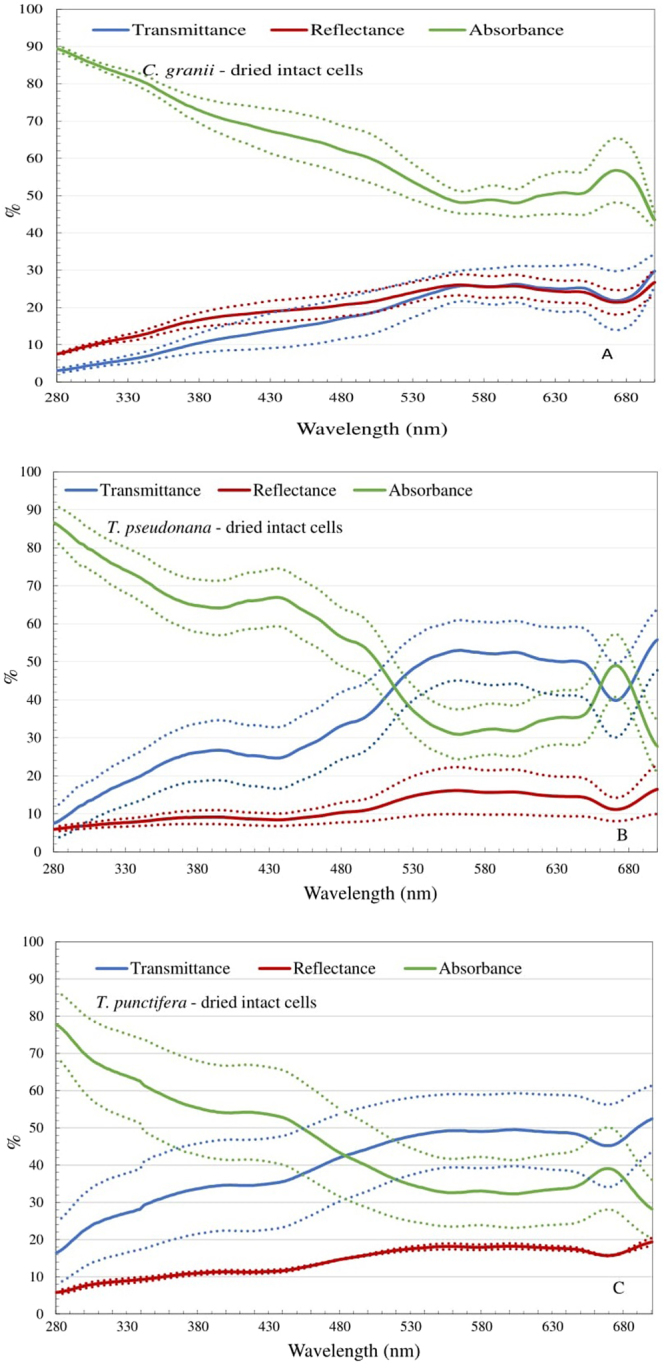
Spectral transmittance (blue), reflectance (red) and absorbance (green) for a coating of dried intact *C. granii* (**A**), *T. pseudonana* (**B**) and *T. punctifera* (**C**) cells. The dotted lines represent the mean value± the standard deviation. The reference spectrum is the UV-transparent quartz silica microscope glass plate with L1 medium on top of it. All the values are normalized to 100% coverage.

As not rinsing the cells for organic material would save time and chemicals in the work process, we also tested the response of dried intact (non-rinsed) cells. The transmittance efficiency of these was 3–11%, 7–26% and 16–34% when the wavelength increased from 280 to 400 nm (UV spectrum) for *C. granii*, *T. pseudonana*, and *T. punctifera*, respectively (Fig. 2). The reflectance was similar for all three species, ranging from 10% to 30% throughout the measured wavelength. The range of reflectance was comparable to the rinsed frustules but the trend was opposite (Figs 1 and 2). For all three species, the absorbance of dried intact cells was massively higher than that of the rinsed frustules in both the visible and UV spectrum (Figs 1 and 2). The absorbance of the dried intact cells at the visible range broadly matched the absorption spectrum of the dominant photosynthetic pigment (Chlorophyll a), with a peak at around 430 nm and a sub-peak at 660 nm^20^. Furthermore, a sub-peak of absorbance was observed at around 480 nm which fits well with the absorption spectrum of diatom carotenoids such as fuxocanthin, diadinoxanthin and diatoxanthin^20^.

### Silica atomic structure

To test whether differences in silica atomic structure could be a cause of the differences in photonic properties between the three species, X-ray scattering analysis was performed on rinsed frustules (Fig. S2 in supplementary information). The wide-angle x-ray scattering (WAXS) curves (Fig. S2A in supplementary information) show no signs of crystallinity in the silica structures of the frustules; thus we conclude that the silica in the frustules of all three species is in an amorphous form. The difference in the height of the broad amorphous peak (at around *d* ≈ 0.4 nm for all the three species, equivalent to 2*θ* ≈ 22, see Fig. S2A) in the WAXS curves is primarily the result of the variable amount of material in the samples. For *C. granii* and *T. punctifera*, another peak is observed at around *d* ≈ 1.5 nm (equivalent to 2*θ* ≈ 5.89, see Fig. S2A) indicating the presence of small smectite (clay) particles in the frustules^21^.

### Frustule nanostructure

**Figure 3:**
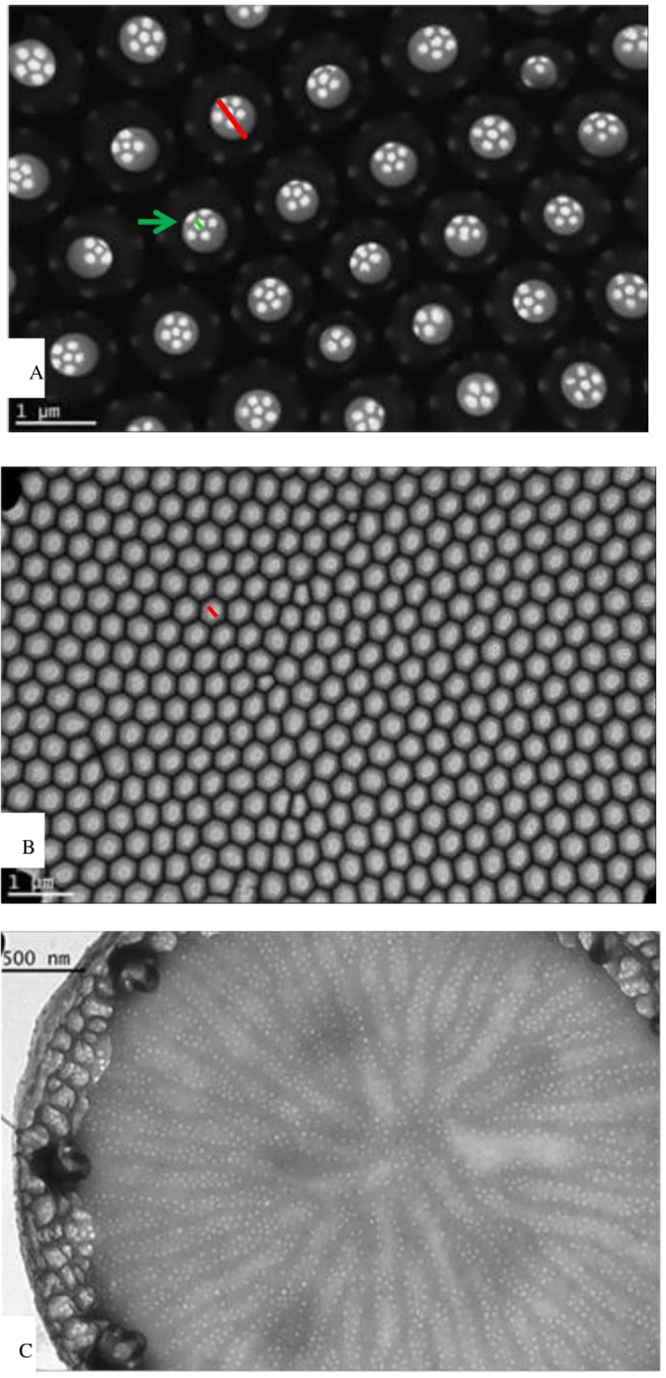
Transmission Electron Microscopy (TEM) viewgraphs of (**A**) *C. granii*, (**B**) *T. punctifera* and (**C**) *T. pseudonana*.

The geometrical arrangement and size-scale of the frustule pores are suggested as one of the critical factors contributing to the UV light extinction effectiveness^13^. For all samples, the porod exponent of small-angle x-ray scattering (SAXS) curves show close to *q*^−4^ decay indicating that the frustule surface of the three species is smooth. *Coscinodiscus granii* is a radial centric diatom with small cribrum pores like a sieve structure on the outer surface (Fig. 3A, green line) and larger pores (foramen: 690.0 ± 80.0 nm, measured by TEM) on the internal surface (Fig. 3A, red line). The SAXS result on *C. granii* can be described simply by a power law and a constant background indicating that even the small cribrum pores in the valve are larger than ca. 100 nm, which is the resolution limit of the SAXS measurements. The frustules of *C. granii* are wedge shaped with a flat valve face (microscopy and SEM observation) and our measurements based on the TEM analyses indicate a diameter of 68.9 ± 21.3 μm and a foramen density within 10 μm of 8.8 ± 0.6 (Table 1). As shown in Fig. 3A, the foramens are regularly distributed on the valve.

**Table 1:** Frustule morphology (frustule diameter, foramen density and foramen diameter) of three diatom species based on TEM and the SAXS modeling Eq. 1.

Diatom species	Frustule diameter (μm)	Foramen density within 10 μm	Foramen diameter (nm)		Small pore (SAXS, nm)
			TEM	SAXS	
*T. punctifera*	97.9 ± 8.4	22.6 ± 1.2	363.5 ± 26.4	—	48.4 ± 0.38
*C. granii*	68.9 ± 21.3	8.8 ± 0.6	690.0 ± 80.0	—	—
*T.pseudonana*	4.2 ± 1.0	203.0 ± 42.2	32.8 ± 7.3	36.6 ± 0.58	15.0 ± 3.04

The valves of *T. punctifera* are almost flat^22^ with a diameter of 97.9 ± 8.4 µm. It has been reported that the larger pores (foramen, Fig. 3B marked by a red line, 363.5 ± 26.4 nm in diameter measured by TEM) are on the outer valves and the sieve pores (48.4 nm in diameter measured by SAXS, Fig. 3B white dots around each foramen) are on the internal surface^22^, which is opposite to *C. granii*. The number of foramen in 10 µm is 22.6 ± 1.2 (Table 1).

Compared with the other two tested diatom species, *T. pseudonana* is much smaller (4.2 ± 1.0 µm, Table 1). It has been reported that their valves are flat or slightly convex with unchambered striae on the valve surface (around 50–70 in 10 µm)^17^. Numerous small pores are distributed within the areolation and the diameter of these is 32.8 ± 7.3 nm (Table 1) based on TEM observation, which is in good agreement with data from the SAXS analysis (36.6 nm, Table 1). The slight difference may be due to the different methodologies: analysing the SAXS curves required fitting a varying number of structural levels and provides mean values; the results obtained from TEM are based on the measurement of randomly selected single valves. An even smaller category of pores, with a diameter of ca. 15.0 nm, was also detected by SAXS but was not obvious in TEM pictures.

### UV transmission and reflection efficiencies by single and double drop-casted layers of rinsed *T. punctifera* frustules

**Figure 4:**
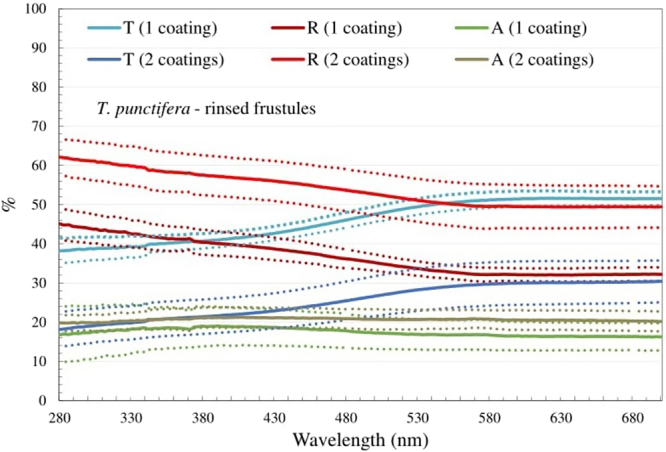
Spectral transmittance, reflectance and absorbance for the one layer (thickness = 18.1 ± 14.7 µm) and two layers (thickness = 36.2 ± 19.6 µm) coated film with rinsed *T.* *punctifera* frustules. The dotted lines represent the mean value± the standard deviation. The reference spectrum is the UV-transparent quartz silica microscope glass plate with nothing on top of it. All the values are normalized to 100% coverage.

Based on the spectral data, *T. punctifera* had the highest UV protection potential and was therefore selected for testing the effect of the thickness of the layer of frustules on the spectral transmission and reflection. Surface topography maps obtained from Infinite Focus Microscope measurement (Fig. S3) showed that the thickness of double drop-casted layer (36.2 ± 19.6 µm) was twice that of the single drop-casted layers (thickness = 18.1 ± 14.7 µm). The spectrum indicated that the transmittance of the double drop-casted layers of rinsed *T. punctifera* was noticeable lower than single drop-casted layers (ca. 55% lower for UV-light), while the shape of the curves were similar throughout the whole measured spectrum (Fig. 4). The reflectance of the double layers was 37% higher (for UV light).

## Discussion

### Light spectra of the rinsed frustules

Similar results to the ones found here have been reported for single valves of *Coscinodiscus wailesii*, i.e. that transmission efficiencies increase with wavelength of visible light in the range from blue (20%) to green (30%) to red (80%)^23^. In rinsed frustule suspensions of six different Antarctic diatoms around 13% to 29% of the total UV-B was absorbed^24^, which is similar to the absorption levels calculated here for *C. granii* and *T. punctifera*. The frustule material itself (silica) absorbs slightly more in the UV-B range than in visible light but does not contribute much to UV-B absorption^12^.

*Yamanaka et al*. showed that a single frustule of *Melosira variance* (a fresh water diatom) absorbed light (from 300 to 800 nm) mainly in the blue wavelength due to a strong interaction between blue light and the nanostructures inside the frustule^14^, providing evidence that the pathway of incident light was wavelength dependent. However, no such obviously higher absorbance in the blue range was observed in our measurements. It is worth noting that *Yamanaka*’s results are based on a single frustule after heating at 500 °C and 600 °C to remove the organic material^14^ and our results are the overall performances of a layer of chemically rinsed frustules. For a single frustule, it is indicated that diffraction plays an important role for light interaction with both foramen and frustule edges^10,11^, while the transmittance and reflectance we measured are absolute overall values which covered all including light scattering and diffraction. It has further been found by simulation that the focusing effect of the frustule of centric diatom is wavelength dependent and no significant confinement within or close to the cells was found in the UV light range^10,16^.

### Intact dried cells

Besides the peaks of absorbance efficiency at 430 nm, 480 nm and 660 nm, which fit the absorbance peaks of chlorophyll a and carotenoids, trace amounts of organic and inorganic material embedded in frustule^25^ and mycosporine-like amino acids in the relatively low concentrations in the algal cytoplasm^26^ could contribute to the higher absorption of dried intact cells in UV range compared with the rinsed frustules as they have been shown to be a source for UV absorption^12,27^. However, long-term effects on the light would be more unpredictable for a layer of intact cells with cell content compared to rinsed frustules due to the ongoing decomposition of the organic material, particularly the photosynthetic pigments. Comparing the two types of material, the intact, non-processed cells have much higher absorption of light and have the advantage that they would be cheaper and less time consuming to process, but the disadvantage of instability in the spectral properties.

### Factors that may influence the UV filtering effects of the rinsed frustules

Molecules such as silaffins, long-chain polyamines, frustulines, cingulines and chitin^28–30^ might contribute to the differences in light propagation and may be so tightly embedded in the silica matrix that they are not completely removed by the methods used to clean frustules of organic material. While we have not found studies that specifically test the method used here, it is a very potent oxidation method^31^ and Romann *et al*.^32^ tested a similar harsh method (hydrogen peroxide) and compared it to a milder method (a combination of a surfactant and a complexing agent) and found that there was no fluorescence signal from organic material after treatment by the harsh method.

The possibility that organic components imbedded in the frustules are not completely removed by the oxidation and that these may influence the spectral properties of rinsed frustules of the three species deserves to be explored for two reasons: Although we expect that the relatively harsh oxidation employed in the present study has probably removed most organic material, other methods used for cleaning diatom valves have been shown to leave remains of organic material in the silica matrix^29,32^ and this possibility is therefore relevant for industrial applications using a range of oxidation methods. The organic templates are species-specific^30^, and might therefore contribute to the differences in UV filtering effect. The other reason is that studies on sponges, which also form silica structures, have shown that the combination of silica and chitin found in the spicules are important for fiber-optical effects in these structures^33^. This might also be important for some photonic features of diatom frustules.

Although the frustules of diatoms are predominantly made of silica and usually characterized as amorphous hydrated silica^10^, small differences in the frustule crystal structure of the different species^21,34^, might contribute to the differences in light propagation throughout the measured wavelength, but our WAXS results indicate that there is no indication of crystalline structure in the frustules of the three species and we could therefore rule out the contribution of differences in the crystalline structure to the differences in UV transmission and reflection. The broad amorphous peak (at around *d* ≈ 0.4 nm, equivalent to 2*θ* ≈ 22, see Fig. S2A) seen in all three species is slighter lower than that reported in previous work (0.43 nm)^21^. This value reflects an average distance in the amorphous silica matrix and is probably influenced by temperature, humidity, water content and possibly other factors as sample preparation, growth conditions, general biological variability and mineral composition.

Apart from the frustule crystal structure, the overall thickness of the frustule is also a critical factor for UV filtering efficiency in the case of single frustules^13^. Similarly, the thickness of the frustule layer may affect the overall UV filtering efficiency. The spectrum results of single and double drop-casted layers of *T. punctifera* showed that a thicker layer leads to higher UV filtering efficiency (Fig. 4). However, although the thickness of the two-drop layer was twice that of the one-drop layer, the change in reflectance was only around 37%. This clearly indicates that although the layer thickness is an important factor, the spectral characteristics of the layer do not scale linearly with thickness. When calculating amounts of diatoms frustules for industrial applications this is a factor, which should be taken into account.

Three photonic phenomena may result in shielding incident light selectively for specific wavelengths: wave-guiding, Bragg scattering and photonic bandgaps (PBG)^11^. Wave guiding phenomena in diatoms are dependent on both the wavelength of incident light and the frustule morphology^7^. If specific conditions are met, Bragg scattering or PBG can be formed where certain wavelengths of light can’t propagate in the materials and are reflected^7,11^. One of the determining factors is the refractive index contrast between material and the surrounding media^23^. The larger index differences are, the higher the possibility is to form Bragg scattering or photonic bandgaps which lead to a higher UV protection efficiency^11^. The diatom frustules themselves have a refractive index of 1.43–1.44^7,11^. In the case of our resultant material with rinsed frustule, the rinsed frustules are drop-casted on light-transparent slides and the Milli-Q water is evaporated. Therefore, the index contrast will be the difference between the frustule (1.43–1.44) and air (1), which is generally not considered high enough to cause PBG under most conditions. When selecting matrix-materials in which the frustules will be embedded to make a durable coating, the role of air should be considered, for example to what degree the matrix will enter into the pores of the frustules and what is the resultant reflective index will be. We do not see resonance behavior (large dips in the spectra) in the spectra, so no photonic bands or Bragg scattering are observed. This can either be because they are not present in the structures or because the effect is hidden within the absolute values measured in these experiments. Another optical phenomenon to consider is Rayleigh scattering^35^. However, the extent of the general increase in reflectance towards shorter wavelengths (Fig. 1(A–C)) does not follow the 1/λ^4^ dependence of Rayleigh scattering.

Other critical factors for the interaction between light and frustules are the pore size and their geometrical arrangement^11,13^. Experimental data^36^ testing the light diffraction pattern behind valves of the centric diatom (*C. wailesii*) was confirmed by simulations^16,37^ which included only the large pores (foramen) (ignoring the small sieve pores) indicating that the large pores play an important role and is a central parameter for light propagation, while the smaller pores apparently are less important for these effects^11^. It has been hypothesized that a pore size of 200–250 nm might be suitable for UV (A and B range) protection purposes^11^ and among the three diatom species tested here, the size of foramen for *T. punctifera* 363.5 ± 26.4 nm comes closest to this range and indeed also shows the highest UV protection efficiency. Both foramen and the inter-distance between pores (450 nm, based on foramen density within 10 μm) of this species have dimensions similar to the wavelength of UV light, and light with wavelengths around these values will interact strongly with the structures, something which is also observed in the spectra. The small pores (48.4 ± 0.38 nm) are below the Rayleigh limit so there might also be a contribution from this (although structural features in the Rayleigh regime could not alone result in such high UV-protection efficiency as observed in Fig. 1C). These combined factors are likely contributing causes behind *T. punctifera* showing the best spectra for UV-protection, with the lowest transmission in the UV ranges.

For the single valves, the optical properties of the external and the internal valve might be different. In addition, where and at which angle the light hits the valves may also play a role for interactions with light. However, when applying a layer of frustules, our data indicate that on average these differences even out. This is seen from the fact that we had very similar patterns in all replicates (for all three species) in spite of completely random orientations (and with this, angle of light incidence) and only slight differences in coverage. An integrating sphere was employed in this measurement and therefore a net-result of transmission, respectively reflection, was obtained. This means that we don’t observe the angle dependence of scattered light but only whether it in the end comes through or not.

## Materials and Methods

### Culture growth

Three diatom species, *Coscinodiscus granii* (strain K-1834), *Thalassiosira pseudonana* (strain K-1282) and *Thalassiosira punctifera* (K-1901) were obtained from the Scandinavian Culture Collection of Algae & Protozoa (now merged with NIVA Culture Collection of Algae). All strains were kept at 15 °C and around 100–150 μmol photons m^−2^s^−1^ (Panasonic FL40ss Enw/37, Japan) with a light/dark cycle of 16 h/8 h without aeration. The growth medium was L1 medium with a salinity of 30 prepared with autoclaved seawater^38^.

### Morphology analysis with TEM

Near the end of the exponential growth stage (around 10 days), a subsample of each of the three diatom species were acid cleaned to remove organic material^39,40^. The cleaned frustule material was drop-casted onto copper grids, and examined using a JEM-1010 (Jeol, Japan) Transmission Electron Microscope (TEM). Frustule diameter, foramen number in 10 µm (foramen density) and the diameter of the foramen were measured on 20–26 frustules from each species and average values were calculated.

### UV light transparency, reflectance and absorbance by spectrophotometer measurements and the thickness of the coated film

Drops (around 10 µl) of concentrated solutions of rinsed frustules (in Milli-Q water, *C. granii*: 240,000 cells/ml; *T. pseudonana*: 3,000,000 cells/ml; *T. punctifera*: 210,000 cells/ml), respectively living cells of each species (in L1 growth medium) were dripped onto the measuring-point (M.P) of a light-transparent (UV-Visible) quartz silica microscope glass plate and dried at room temperature. Two drops were needed until the M.P (8 mm in diameter, Fig. S1 in supplementary information) was nearly completely covered. Microscope pictures were taken using an Olympus GX41 light microscope (Japan) to calculate the layer coverage of the measuring-point as shown in Fig. S1. Transmittance (T) and Reflectance (R) spectra (from 280 to 700 nm) were measured using a Shimadzu UV-2600 spectrometer (Japan) with build-in light sources and integrating sphere. The reason we start the measurement from 280 nm (UV B) is that almost all UV light below 280 nm (UV C) is absorbed by the ozone layer and therefore does not reach to earth’s surface and its contribution is therefore insignificant^41^.

Absorbance (A) was calculated based on the following equation: A = 100 – (T + R). All the measurements were normalized to 100% coverage. The surface topography of the layer was measured using an Alicona Infinite Focus Microscope (IFM, Alicona Imaging GmbH, Graz, Austria). The technique allows capturing 3D morphology and depth information at maximum magnification (100×), a lateral resolution down to 400 nm and vertical resolution of 10 nm. The IFM measurement was analyzed with a software processor package (Scanning Probe Image Processor, SPIP^TM^, Image Analysis A/S) to characterize the thickness of the coated film. For thickness measurement, 1 drop (around 10 µl) of concentrated solution of *T. punctifera* rinsed frustules was dripped onto a UV-transparent quartz silica microscope glass plate and dried at room temperature to get one layer samples. The same procedure was repeated one more time to make the two layers samples. All experiments were performed in triplicate except for the measurement for single drop-casted layers (duplicate).

### Wide angle X-ray scattering setup and modeling

Small- and Wide-angle X-ray scattering (SAXS/WAXS) profiles were measured using a GANESHA instrument from SAXSLAB (Lyngby, Denmark). The instrument uses a Rigaku (Rigaku-Denki, Co., Tokyo, Japan) 40 W micro-focused Cu-source producing X-rays with a wavelength of λ = 1.54 Å detected by a moveable Pilatus 300k pixel-detector from Dectris (Baden, Switzerland) allowing different length scales to be measured. The two-dimensional scattering data were azimuthally averaged and corrected for detector inhomogeneities using standard reduction software (SAXSGUI). The radially averaged intensity *I* is given as a function of the scattering vector *q* = 4π sin *θ/λ*, where λ is the wavelength and 2*θ* is the scattering angle. Two instrument settings were used: a SAXS setting covering a *q*-range from 6.3·10^–3^ Å^−1^ to 0.27 Å^−1^ and a WAXS setting covering a *q*-range from 0.1 to 2.45 Å^−1^ corresponding to an upper 2*θ* value of 35 degrees or 2.56 Å.

The SAXS data are analyzed using the unified Beaucage model^42^. In the model the scattering intensity of *N* structural levels with radii of gyrations *Rg* is described by a sum with terms for each level given by

1 $$I(q)={G}_{exp}(-{q}^{2}{R}_{g}^{2}/3)+B{\{{[erf(q{R}_{g}/\sqrt{6})]}^{3}/q\}}^{P}$$

where *G* and *B* are constants and *P* the Porod exponent. The first term is the Guinier term and the second account for Porod (surface) scattering and the two are smoothly combined via the error-function term. In the final model a small constant background is added to account for the flat scattering from the thin mica windows the samples were mounted between. Data and model agreement was optimized using standard least-squares fitting done in Matlab.

## Electronic supplementary material

Supplementary information
