# Supplementary material for: The UV filtering potential of drop-casted layers of frustules of three diatom species
Source: Sci Rep. 2018 Jan 17;8:959. doi: 10.1038/s41598-018-19596-4 (PMC5772478; doi:10.1038/s41598-018-19596-4)
Supplement: Supplementary file 1 — Supplementary information [file 41598_2018_19596_MOESM1_ESM.pdf]

The UV filtering potential of drop-casted layers of frustules of three diatom species

Yanyan Su[1*], Torben A. Lenau[2], Emil Gundersen[1], Jacob J. K. Kirkensgaard[3], Christian Maibohm[4], Jérôme Pinti[5], Marianne Ellegaard[1]

[1] Department of Plant and Environmental Sciences, University of Copenhagen, Thorvaldsensvej 40, 1871 Frederiksberg, Denmark

[2] Department of Mechanical Engineering, Technical University of Denmark, Produktionstorvet, Building 426, 2800 Kongens Lyngby, Denmark

[3] Niels Bohr Institute, University of Copenhagen, 2100 Copenhagen, Denmark

[4] International Iberian Nanotechnology Laboratory, Avenida Mestre José Veiga s/n, 4715-330 Braga, Portugal

[5] DTU Aqua, National Institute of Aquatic Resources, Technical University of Denmark, Kemitorvet, building 202, 2800 Kongens Lyngby, Denmark

[*] Corresponding author: Tel: +45 35320437. Email: yanyansu@plen.ku.dk.

Supplementary Information 5 Pages, 1 Table and 3 Figures

Table S1 The percentage of the coverage for three tested diatom species (two layers, dried intact cells and rinsed frustules) and for rinsed frustule of *T. punctifera* (one layer)

	Thalassiosira punctifera (one and two layers)			Coscinodiscus granii (two layers)		Thalassiosira pseudonana (two layers)	
	Dried intact cells (two layers)	Rinsed frustules (two layers)	Rinsed frustules (one layer)	Dried intact cells	Rinsed frustules	Dried intact cells	Rinsed frustules
Coverage rates (%)	92±3	97±1	95±2	99±1	94±2	99±1	91±5

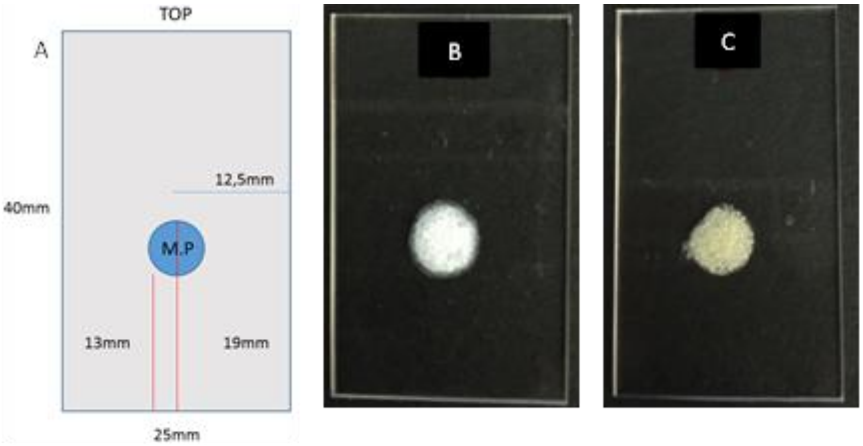

Fig. S1 Schematic diagram (A) of the UV-transparent quartz silica microscope glass with Measuring-Point (M.P); photography of UV-transparent quartz silica microscope glass coated with rinsed frustule (B) and dried intact cells (C).

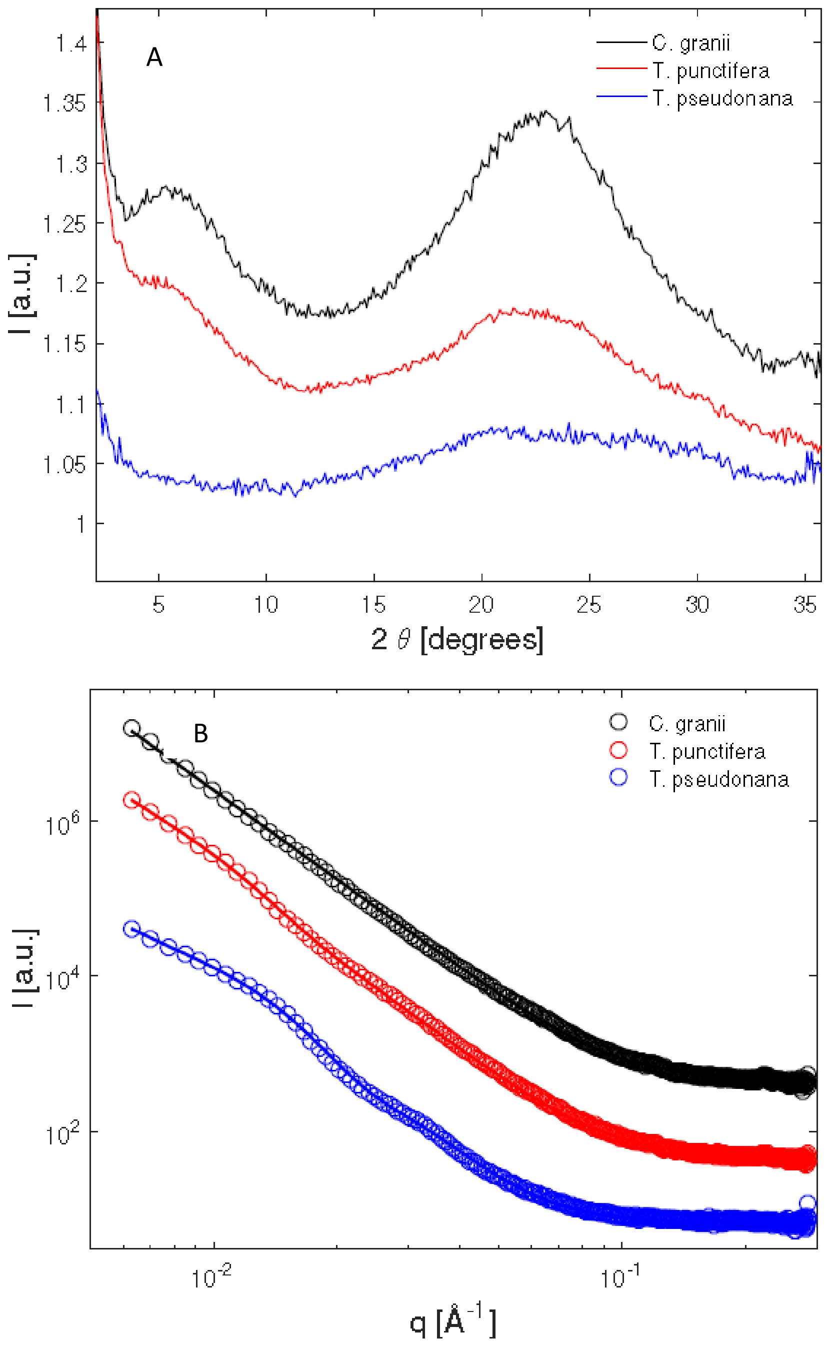

Fig. S2 WAXS (A) and SAXS (B) data from the frustules of *C. granii*, *T. pseudonana* and *T. punctifera*. Full lines in B are fits to Equation 1. Y-axis is in [a.u.] arbitrary units for visualization only.

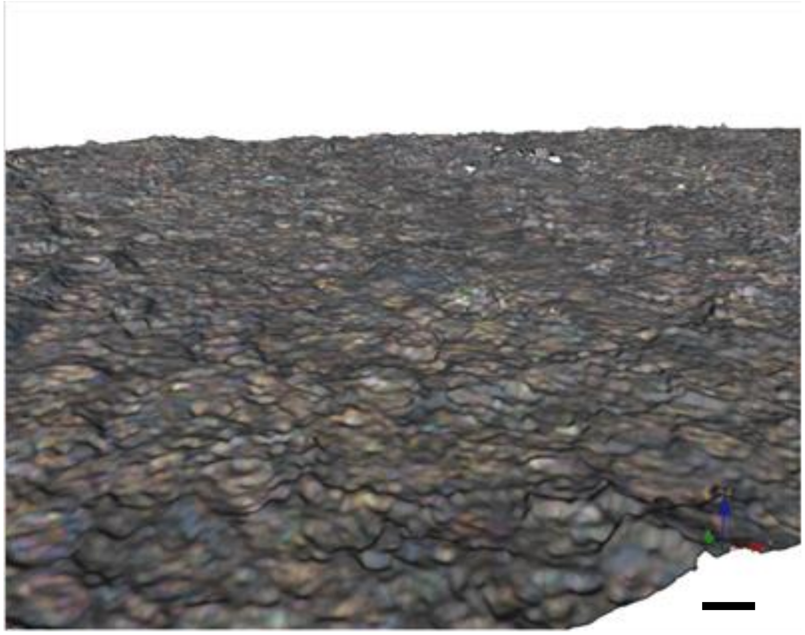

Fig. S3 Surface topography maps of *T. pseudonana* (single drop-casted layers) taken by Infinite Focus Microscope (Scale bar: 1 mm).
